# Is comorbidity in adolescence a predictor for adult low back pain? A prospective study of a young population

**DOI:** 10.1186/1471-2474-7-29

**Published:** 2006-03-16

**Authors:** Lise Hestbaek, Charlotte Leboeuf-Yde, Kirsten O Kyvik

**Affiliations:** 1 The Back Research Center, Backcenter Funen, Part of Clinical Locomotion Science, University of Southern Denmark; 2 The Danish Twin Registry, University of Southern Denmark, Odense, Denmark

## Abstract

**Background:**

It has previously been shown that low back pain (LBP) often presents already in the teenage years and that previous LBP predicts future LBP. It is also well documented that there is a large degree of comorbidity associated with LBP, both in adolescents and adults. The objective of this study is to gain a deeper insight into the etiology of low back pain and to possibly develop a tool for early identification of high-risk groups. This is done by investigating whether different types of morbidity in adolescence are associated with LBP in adulthood.

**Methods:**

Almost 10,000 Danish twins born between 1972 and 1982 were surveyed by means of postal questionnaires in 1994 and again in 2002. The questionnaires dealt with various aspects of general health, including the prevalence of LBP, classified according to number of days affected during the previous year (0, 1–7, 8–30, >30). The predictor variables used in this study were LBP, headache, asthma and atopic disease at baseline; the outcome variable was persistent LBP (>30 days during the past year) at follow-up. Associations between morbidity in 1994 and LBP in 2002 were investigated.

**Results:**

LBP, headache and asthma in adolescence were positively associated with future LBP. There was no association between atopic disease and future LBP. Individuals with persistent LBP at baseline had an odds ratio of 3.5 (2.8–4.5) for future LBP, while the odds ratio for those with persistent LBP, persistent headache and asthma was 4.5 (2.5–8.1). There was a large degree of clustering of these disorders, but atopic disease was not part of this pattern.

**Conclusion:**

Young people from 12 to 22 years of age with persistent LBP during the previous year have an odds ratio of 3.5 persistent LBP eight years later. Both headache and asthma are also positively associated with future LBP and there is a large clustering of LBP, headache and asthma in adolescence.

## Background

Chronic low back pain (LBP) is a major socio-economic burden for both society and individuals. In Denmark, musculo-skeletal disorders, of which LBP was the most common, was the reason for 50% of all disorders, classified as "work-related", in the year of 2000 [1]. In 2002, it was the second-most common reason for disability pension, responsible for 22% of all disability pensions [1]. Furthermore, the cost of long-term sick leave due to musculo-skeletal disorders increased from 1987 to 2000 in Denmark [2].

Unfortunately, we are unable to predict chronic LBP at an early stage. Several risk factors for chronicity, such as psychological distress or depressive mood [3], poor general health [4-6], and work-related factors [7,8], have been identified. These findings are based on studies of adult patient-populations, although it has been shown that more than half of the population experience their first attack of LBP before the age of 20 [9]. Thus, studies of adults are not likely to include inception cohorts and their results cannot be used to aid in the design of *primary *prophylactic efforts. Therefore, we find it important to shift focus from risk factors associated with LBP in adult life to *early predictors *for LBP. Both our group and others have previously shown LBP in adolescence to be a major predictor for later LBP [10-12]. Our group has also demonstrated strong cross-sectional associations between LBP in adolescence and the presence of various other diseases [13]. This investigation aims to investigate how LBP and other diseases in young people are associated with later persistent LBP.

## Methods

### Data sources

The Danish Twin Register is the most comprehensive population based twin register in the world, spanning a period of more than 100 years. The twins of interest for this study were born between 1972 and 1982. They were identified through the Danish civil registration system and represent 95% of the twins born during that period. These twins can be regarded as representative of the general population since they have the same mortality rate [14] and the same prevalence of various diseases as the population at large, such as insulin dependent diabetes [15], hand eczema [16], asthma and allergic rhinitis [17], and LBP [18]. Details about ascertainment etc. are provided elsewhere [19]. In 1994, when the twins were 12 to 22 years old, comprehensive questionnaires were sent to those twins who previously had agreed to participate in future studies (96%). The questionnaires contained questions on disease, health and health-related behaviour. Similar questionnaires were sent to the same population in 2002, when they were 20–30 years of age. The outcome-variable of interest for this paper was the number of days with LBP during the year prior to follow-up. The same variable at baseline was used as one of the covariates. The exact wording of the question was: "How many days have you *altogether *had trouble with the lower part of your back during the past year?". The additional, relevant information was based on the following questions: Have you ever had any of the following: diabetes, asthma, atopic dermatitis, hay fever, wheezing respiration from colds, exertion, or from contact with animals or pollen, shortness of breath when walking, long periods (> a month) with cough and phlegm, dry cough not connected to colds, juvenile rheumatoid arthritis, rheumatoid arthritis, migraine, headache with nausea, headache associated with photophobia/phonophobia, temporary visual impairment followed by headache, or severe ocular pain. The respondents also reported number of days with headache during the past year. The exact wording is available from the authors. Validation: The questions regarding LBP were modelled on the Nordic Back Pain questionnaire [20], which has been validated previously [21]. In a previous study, analyses of validity were performed on the data from the 1994-omnibus by cross-tabulating LBP-days with LBP-ever and found to be satisfactory [13]. Similarly, the reliability of LBP-questions from the 2002-omnibus has previously been considered to be satisfactory through identification of logical errors [10].

### Representativeness

Responders and non-responders at follow-up were compared with regard to age, gender, LBP-status and all the covariates at baseline.

### Variables

Number of days with LBP during the past year was transformed into persistent LBP ('LBP-long'), defined as LBP for more than 30 days during the previous year, and LBP at all ('LBP-all'), defined as LBP for one day or more during the previous year. Persistent (or recurrent) LBP is the most interesting outcome variable, both from a socio-economic perspective and from an individual's perspective, since brief/transient episodes of LBP do not influence the professional or the social life of the person to any large extent. Therefore, we chose persistent LBP ('LBP-long') as outcome variable.

Because the focus of this investigation is on self-reported symptoms, the covariates were grouped into three broad categories of symptoms:

• The first group covers those with asthma-like symptoms with or without a diagnosis of asthma ('asthma'): It consists of those who reported having at least one of the following: asthma, wheezing breath from colds, exertion, or from contact with animals or pollen, shortness of breath when walking, long periods (> a month) with cough and phlegm, and dry cough not connected to colds.

• The second group includes people with possible atopic disease, but no evidence of asthma ('atopic disease'), and consists of those who answered that they had atopic dermatitis and/or hay fever, but without asthma. It should be noted that according to these definitions asthma and atopic disease do not overlap.

• The third group covers all types of headache, regardless of etiology ('headache') and includes those who answered positively to at least one of the following: migraine, headache with nausea, headache with photophobia/phonophobia, temporary visual impairment followed by headache, severe ocular pain. Finally, the respondents noted the number of days with headache the past year. Analog to LBP, this was transformed into persistent headache ('headache-long'), defined as headache for more than 30 days during the previous year, and headache at all ('headache-all'), defined as headache for one day or more during the previous year.

There were a total of 26 individuals (0.3%) with diabetes and 30 (0.3%) with rheumatoid arthritis at baseline. Thus, both diabetes and rheumatoid arthritis were too rare to do any meaningful analyses and were not analysed further.

### Analyses

The associations between the outcome variable and the individual independent variables (asthma, atopic disease, headache and LBP) were investigated through bivariate logistic regression analyses. Confidence intervals of 85% were used to decide inclusion or exclusion in the multivariate analyses, since a statistical significance of 0.05 is too stringent. Variables meeting this cut-off point may still influence the effect of the other variables. Since the prevalence of LBP increases with age and is higher in females, age and sex were forced into all models.

Thereafter, those variables found to be statistically significant at the 0.15-level in the bivariate analyses were cross-tabulated to assess the size of the subgroups and the combinations between disorders. The same variables were then included in multivariate logistic regression analyses. Possible interactions between predictor-variables were investigated, which was possible because of the large sample size. Odds ratios were calculated for two models: one including the more lenient definitions of LBP and headache and another including the persistent types of LBP and headache. Age and gender were forced into the models and if they were significant, they were removed from the model and results were stratified instead. Finally, to gain an overview of the practical value of the results, prevalence estimates of persistent LBP at follow-up were presented for the relevant subgroups.

Post-hoc analyses: Due to concern that "atopic disease" might mask a possible effect of one the two disorders (atopic dermatitis and hayfever), separate bivariate analyses were performed for these two disorders.

All analyses were done using the STATA 8.0 statistical software package. The cluster-option as provided by STATA was applied to account for dependency between twins and p-values of 0.05 or less were considered to be significant.

## Results

### Study sample and representativeness

The study sample is described in Table 1 for both baseline and the 8-year follow-up. The age distribution was the same for responders and non-responders, but there were 4% more females at follow-up than in the baseline sample. There were no differences between the baseline sample and the follow-up sample with regard to the prevalence estimates of LBP, headache, asthma or atopic disease.

### Bivariate analyses

The bivariate analyses (with age and sex included in the model) showed all the potential predictor variables to be statistically significantly associated with persistent LBP at follow-up, with the exception of atopic disease. The strongest predictors were persistent LBP and persistent headache at baseline. The exact estimates are shown in Table 2.

To investigate whether the associations between a certain disorder and future LBP was merely a measure of this disorder's association with LBP at baseline (bearing in mind the predictive value of LBP on future LBP), we repeated the bivariate analyses in a subsample excluding the 588 individuals with persistent LBP at baseline (Table 3).

The estimates were almost the same as for the whole group, indicating some degree of increased risk associated with headache and asthma, despite LBP-status. There were no associations between atopic disease in youth and later LBP, and the prevalence of LBP, headache and asthma were not statistically different between subjects with and without atopic disease (p = 0.123–0.156). Thus, atopic disease does not seem to be associated with any of the other covariates and was not included in the multivariate analyses.

### Description of subgroups

To gain an overview of the various subgroups, the size of these are listed in Table 4. This shows, that headache is by far the most common disorder. Comparison of the percentages in Table 4 also illustrates the large degree of comorbidity.

### Multivariate analyses

Despite the large overlap between disorders (comorbidity), none of the interaction-terms between the three predictor-variables (LBP, headache and asthma) were found to be statistically significant, and therefore the multivariate models were constructed without inclusion of interactions. Age was not significant, whereas there was a positive association with female gender. Therefore, the results were stratified for gender. The results of the two models – 1) one with all types of LBP and headache (LBP-all and headache-all) and 2) one with persistent LBP and headache (LBP-long and headache-long) – are shown in Table 5. This illustrates no significant difference between the two sexes, but significantly higher odds ratios in the model containing the persistent disorders. For the combination of persistent LBP, persistent headache and asthma the odds ratio is 4.5 (2.5–8.1). If asthma is ignored, the odds ratio is similar: 4.6 (2.9–7.4). Of the 86 individuals with both persistent LBP and persistent headache, 50 (58%) also have asthma.

### Prevalence of persistent LBP at follow-up for the various subgroups

**Figure 1:**
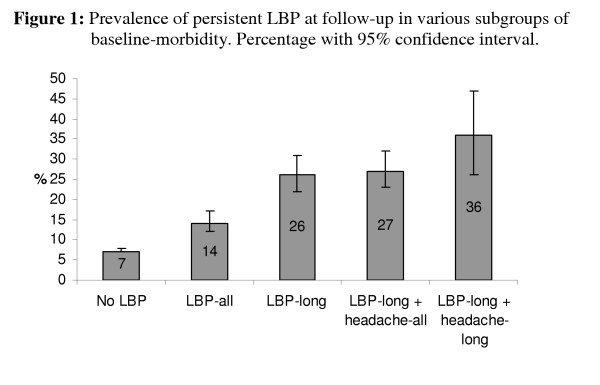
Prevalence of persistent LBP at follow-up in various subgroups of baseline-morbidity. Percentage with 95% confidence interval.

To illustrate the increasing prevalence of LBP, as the subjects' morbidity increases, we calculated the prevalence of persistent LBP in the various subgroups of baseline-morbidity. The overall one-year prevalence of persistent LBP in 2002 was 10%. For those with no LBP in 1994, the one-year prevalence of persistent LBP in 2002 was 7%, for those with any LBP at all 8 years previously, it was 14%, and if this LBP was persistent, the prevalence was 26%. If the persons in addition to persistent LBP in 1994 also suffered from headache, the resulting one-year prevalence of persistent LBP at follow-up was 27%, and if this headache was long-lasting, the corresponding prevalence was 36%. This stepwise effect is illustrated in Figure 1.

### Post-hoc analyses

Post-hoc analysis of the association between atopic dermatitis at baseline and persistent LBP at follow-up revealed an odds ratio of 0.99 with an 85% confidence interval of 0.93 to 1.05. Similar analysis of hayfever gave an odds ratio of 1.04 with an 85% confidence interval of 0.98 to 1.10. Thus, none of these two conditions were included in the multivariate analyses.

## Discussion

The major strengths of our cohort are the large sample size and the age of the subjects. Monitoring the health of such a large sample from adolescence into adulthood can provide much needed clues to early prevention. Furthermore, the cohort is representative of the total Danish population and can be followed also in future years. As mentioned earlier, LBP has previously been shown to be a strong predictor for future LBP [10-12] and associations between LBP and various other disorders have often been shown [4-6]. However, we are not aware of any other studies investigating how other aspects of health in young populations are associated with later LBP.

Unfortunately, the response rate fell to 68% at follow-up, probably due to a large increase in the length of the questionnaire. A comparison of responders and non-responders shows a somewhat higher proportion of females at follow-up, but no differences in the independent or the dependent variables. Therefore, the lower response rate is unlikely to have any bearing on our results, when stratified for gender.

We found LBP, headache and asthma to be statistically significant predictors for future persistent LBP in bivariate analyses with persistent LBP demonstrating by far the strongest association. The multivariate analyses showed that there was in fact a statistically significant effect of all three disorders, meaning that the results from the bivariate analyses were not just a result of baseline-comorbidity. The estimated odds ratio for persistent LBP at follow-up was higher if the individual had LBP-long, headache-long and asthma than if only LBP-long was included in the model (4.5 vs. 3.5), but this difference was not statistically significant and at the same time, by reducing the size of the high-risk group, the number of identified cases was strongly reduced. Therefore, as a screening tool, simply asking about LBP is more efficient than to include all three variables in this question. Although persistent LBP is the strongest predictor, we do not know whether LBP is the key etiological issue, and therefore cannot conclude that comorbidity does not matter. With the large degree of clustering of disorders in some individuals, it is possible that some other underlying factor is the main reason behind the predictive value of LBP. The large overlap of disorders, but no effect-modification (interaction) at baseline, could indicate some common cause for the various disorders. There could be some underlying frailty, rendering some individuals more susceptible to various disorders. The important aspect of this study is that if such a frailty exists, it persists into adulthood. Such a 'common cause' or underlying frailty could be genetically determined, due to social vulnerability or based on morphology, physiology or certain psychological traits. If such a frailty can be detected early in life, many later problems might be prevented or diminished.

Obviously, with a prevalence of persistent LBP of only 26% in the subgroup with persistent baseline LBP, this single variable is a crude predictor. However, as it requires only one simple question, which could be easily incorporated in routine medical examinations, we feel it could be a sensible way to start. Those with persistent LBP at an early age can then be submitted to close monitoring. This is a subgroup of only 6% of the entire cohort, with the remainder of the cohort having a rather low risk (prevalence of 9%) and thus, resources could be allocated to a group with a risk that is much higher than average. The relevance of this is accentuated, when considering the age of the cohort – most of them have not yet entered the workforce. Since some professions are associated with higher risk of LBP than others [22], an evaluation of a young person's risk of future LBP should be part of good career-counselling programmes. However, in this work it must also be emphasized that despite the significantly increased odds of future LBP in this group, two thirds of these 'at risk' did *not *have persistent LBP at follow-up.

## Conclusion

Young people from 12 to 22 years of age with persistent LBP during the previous year have an odds ratio of 3.5 for persistent LBP eight years later. Although both headache and asthma are positively associated with future LBP, adding information about these other disorders does not help in identifying high-risk groups of persistent LBP. The reason being a large degree of clustering of disorders in these young individuals. This indicates an underlying frailty or common cause for the various disorders which continues into adulthood.

## Competing interests

The author(s) declare that they have no competing interests.

## Authors' contributions

LH and CL-Y conceived of the study, and participated in its design. LH made the statistical analyses and drafted the manuscript. KOK participated in the design of the study. All authors read and approved the final manuscript.

## Pre-publication history

The pre-publication history for this paper can be accessed here:



## Figures and Tables

**Table 1:** Description of sample and comparison of baseline- and follow-up-sample.

	**Baseline**	**Follow-up**
Responders, n (%)	9,600 (84%)	6,554 (57% of the original sample, 68% of the baseline sample)
Age at baseline, mean (range)	17.27 (11–22)	17.38 (11–22)
Female/male, n (%)	4,946 (52%)/4,654 (48%)	3,682 (56%)/2,868 (44%)
LBP-long 1994 (LBP>30 days past year), n (%)	588 (6%)	401 (6%)
LBP-all 1994 (LBP>0 days past year), n (%)	3,223 (34%)	2,322 (35%)
Number of days with LBP 1994, mean (SD)	10.46 (38.37)	10.80 (38.78)
Headache-long	591 (7%)	414 (7%)
Headache-all	8.266 (86%)	5.635 (86%)
Asthma	2.685 (28%)	1.785 (27%)
Atopic disease	998 (10%)	707 (11%)

**Table 2:** Bivariate logistic regression analyses of associations between health status at baseline and persistent LBP at follow-up as well as prevalence of persistent LBP at follow-up within the respective groups. Age and sex were included in the model.

**Morbidity 1994**	**OR for LBP-long 2002 (95%/85% confidence interval)**	**Prevalence of LBP-long 2002 (95% confidence interval)**
LBP-long	3.53 (2.75–4.52)/(2.94–4.23)	26% (22–31%)
LBP-all	1.98 (1.67–2.36)/(1.75–2.25)	14% (12–15%)
Headache-long	2.12 (1.62–2.79)/(1.74–2.59)	20% (16–24%)
Headache-all	1.56 (1.17–2.08)/(1.26–1.93)	10% (10–11%)
Asthma	1.44 (1.21–1.71)/(1.26–1.63)	12% (11–15%)
Atopic disease	1.04 (0.80–1.36) / (0.87–1.27)	10% (8–13%)
All	Not applicable	10% (9–10%)

**Table 3:** Bivariate logistic regression analyses of associations between health status at baseline and LBP at follow-up as well as prevalence of persistent LBP at follow-up within the respective groups. Based on a sample excluding individuals with persistent LBP at baseline. Age and sex were included in the model.

**Morbidity 1994**	**OR for LBP-long 2002 (95% CI)**	**Prevalence of LBP-long 2002 (95% CI)**
Headache-long	2.52 (1.56–4.07)	14% (9–21%)
Headache-all	1.40 (1.04–1.88)	9% (8–10%)
Asthma	1.34 (1.10–1.62)	11% (9–12%)
Atopic disease	1.08 (0.82–1.43)	9% (7–12%)
All	NA	9% (8–9%)

**Table 4:** Cross-tabulation of the independent baseline-variables (n = 9,600). Percentages calculated across rows (i.e. within diagnostic subgroups).

**n (% of total sample)**	**LBP**	**LBP-long**	**Headache**	**Headache-long**	**Asthma**
**LBP-all **3,223 (34%)		588 (18%)	2,974 (92%)	334 (10%)	1,244 (39%)
**LBP-long **588 (6%)	588 (100%)		559 (95%)	134 (23%)	287 (49%)
**Headache-all **8,266 (86%)	2,974 (36%)	559 (7%)		591 (7%)	2,446 (30%)
**Headache-long **591 (6%)	334 (57%)	134 (23%)	591 (100%)		269 (46%)
**Asthma **2,685 (28%)	1,244 (46%)	287 (11%)	2,446 (91%)	269 (10%)	

**Table 5:** Multivariate logistic regression analyses of associations between health status at baseline and persistent LBP at follow-up. Stratified for gender and adjusted for age.

	**Females OR (95% CI)**	**Males OR (95% CI)**
**Model 1**		
LBP-all, n = 3,223	1.78 (1.45–2.23)	2.04 (1.50–2.79)
Headache-all, n = 8,266	1.74 (1.08–2.79)	1.14 (0.78–1.66)
Asthma, n = 2,685	1.36 (1.10–1.69)	1.11 (0.80–1.53)
LBP-all + headache-all + asthma, n = 1,189	1.87 (1.46–2.39)	1.85 (1.25–2.75)
**Model 2**		
LBP-long, n = 588	3.55 (2.60–4.83)	2.54 (1.53–4.20)
Headache-long, n = 591	1.55 (1.13–2.11)	2.40 (1.21–4.74)
Asthma, n = 2,685	1.37 (1.10–1.70)	1.12 (0.80–1.57)
LBP-long + headache-long + asthma, n = 81	4.22 (2.27–7.82)	6.56 (1.19–36.35)
